# Comprehensive analysis of individual pulp fiber bonds quantifies the mechanisms of fiber bonding in paper

**DOI:** 10.1038/srep10503

**Published:** 2015-05-22

**Authors:** Ulrich Hirn, Robert Schennach

**Affiliations:** 1 Institute of Paper, Pulp and Fibre Technology, Graz University of Technology, Inffeldgasse 23, 8010 Graz; 2 Institute of Solid State Physics, Graz University of Technology, Petersgasse 16, 8010 Graz; 3 CD-Laboratory for Surface Chemical and Physical Fundamentals of Paper Strength, Graz University of Technology, Petersgasse 16, 8010 Graz

## Abstract

The process of papermaking requires substantial amounts of energy and wood consumption, which contributes to larger environmental costs. In order to optimize the production of papermaking to suit its many applications in material science and engineering, a quantitative understanding of bonding forces between the individual pulp fibers is of importance. Here we show the first approach to quantify the bonding energies contributed by the individual bonding mechanisms. We calculated the impact of the following mechanisms necessary for paper formation: mechanical interlocking, interdiffusion, capillary bridges, hydrogen bonding, Van der Waals forces, and Coulomb forces on the bonding energy. Experimental results quantify the area in molecular contact necessary for bonding. Atomic force microscopy experiments derive the impact of mechanical interlocking. Capillary bridges also contribute to the bond. A model based on the crystal structure of cellulose leads to values for the chemical bonds. In contrast to general believe which favors hydrogen bonding Van der Waals bonds play the most important role according to our model. Comparison with experimentally derived bond energies support the presented model. This study characterizes bond formation between pulp fibers leading to insight that could be potentially used to optimize the papermaking process, while reducing energy and wood consumption.

Paper is a composite material that has been used for a long time. Currently, the majority of industrial pulp fibers are manufactured from wood. The process of papermaking has remained basically intact since its beginning. A dilute suspension of fibers in water is prepared and evenly distributed on a fine mesh^1^. The water is removed through the mesh and the remaining fiber mat is pressed and dried.

The fiber surfaces, consisting of cellulose, hemicelluloses and lignin in varying compositions, form stable bonds thus creating a composite material with remarkable strength per square meter. The salient feature of papermaking is that the fibers bond to each other without any glue or adhesive material added to the suspension. This bonding is fully reversible, which makes paper an easily recyclable material. Upon immersion in water with concomitant stirring, paper disintegrates into its component fibers, the resulting suspension can be reused in the papermaking process.

The fibers in paper bond to each other by six different mechanisms: interdiffusion, mechanical interlocking, capillary forces, Coulomb forces, hydrogen bonding and Van der Waals forces. Reviews on fiber-fiber bonding and its mechanisms are given by numerous authors^2^,^3^. The strength of non-woven materials, i.e. paper, depends on the strength of these component fibers and the strength of the bond between these fibers. For a given sheet, one of these two is the factor limiting the material strength, as described by the Page equation^4^. Besides modifying network properties^5^, e.g. by densification of the paper, the bond strength is improved via the *specific* bond strength, i.e. the strength per unit area. In this study, we only use specific bond strength given in bond energy per unit area.

Several publications have been addressing qualitative and sometimes quantitative analysis of individual bonding mechanisms. To our knowledge there is no publication quantifying all bonding mechanisms. Therefore, the focus of this paper is on providing the big picture, analyzing all mechanisms quantitatively and comparing the resulting bonding energy to measured data.

## Sample material

In all experiments, we used an industrial unbleached softwood Kraft pulp (Mondi Frantschach, Austria). The pulp was a mixture from spruce and pine (mass ratio about 80/20). It had been cooked to a Kappa number of 42, and it was once dried and unbeaten.

## Results

**Figure 1:**
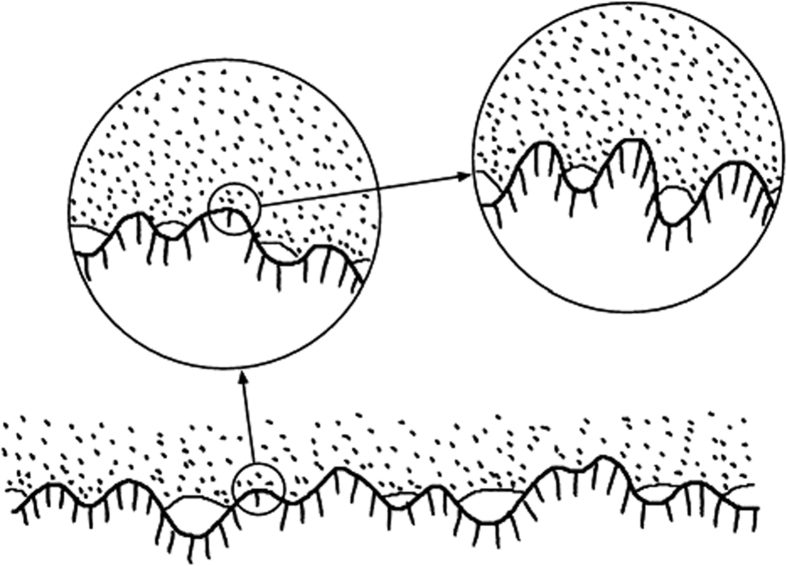
Pictorial schematic between two rough surfaces of pulp fibers showing areas with and without molecular contacts^9^. These surfaces have contacts on all length scales. Regions that seem to have full contact may be separated under larger magnification.

The inter-molecular bonding mechanisms, i.e. hydrogen bonding, Van der Waals forces and Coulomb forces, only become relevant if molecular contact - a distance closer than ~300 Å - is established. Thus the area of molecular contact is the most important factor determining the inter-fiber bond strength because it is a multiplier to the bonding obtained by inter-molecular mechanisms. A previous study provides details on the area in molecular contact of fiber-fiber bonds^6^. Usually the area in molecular contact is different from the optically bonded area (Fig. 1). Unbonded regions with a surface distance smaller than half the wavelength of light (<2000 Å) cannot be detected optically. Regions that seem bonded optically may turn out to be partially unbonded under larger magnification.

Capillary forces establish molecular contacts between the fiber surfaces during drying^3^,^7^,^8^. The key point is that the capillary forces are pressing the surfaces together^9^. This creates contact due to plastic, viscoelastic, and elastic deformation of the fiber surfaces. The degree of molecular contact between the fiber surfaces initially depends on the surface roughness - smooth surfaces permit a high degree of contact. Contact depends on a highly deformable fiber surface in the elastic (low E-modulus) and plastic (low indentation hardness) regime. Recently, a quantitative contact mechanics model describing the development of molecular contact for soft surfaces due to capillary forces was reported^9^,^10^.

Using contact mechanics simulations and experimental evidence^6^,^10^ we previously found that the area in molecular contact in dry paper is close to full contact (about 90% of the optically bonded area). This result is counter-intuitive; however, for soft surfaces this is not uncommon^11^. The wet fiber surface is known to have an extremely smooth and soft^12^,^13^, hydrogel-like structure^14^. The upper limit for the area in molecular contact is full contact^6^. Assuming that there might be an error by a factor of three in the results, the lower limit is 30%.

Cellulose fibers in water are highly swollen. They form a soft hydrogel layer on their surface^14^. The formation of the gel layer enables mutual migration of cellulosic polymers into the opposing surface. Interdiffusion refers to the migration of these molecules, usually polymers, from one fiber into the other. Interdiffusion is believed to be a key mechanism of fiber-fiber bonding^3^,^15^,^16^. It improves the bond strength by increasing the available contact area for the inter-molecular bonding forces. Thomson et al. have adapted Fluorescence Resonance Energy Transfer (FRET) microscopy to study the degree of bonding between cellulosic fiber-fiber surfaces^17^,^18^,^19^. For individual softwood pulp fiber-fiber bonds, they found a 60% increase in molecular contact due to rewetting and repressing of the bonds^18^, which they attributed to interdiffusion of the surface molecules.

Theoretically, the degree of interdiffusion is limited only by the polymer chain length, which is high for cellulosic fibers. For our calculations, we assume that the maximum contribution from interdiffusion is a doubling of the area for molecular interactions. This is a first estimate, based on the experience of the 60% increase in molecular contact after rewetting and repressing of fiber-fiber bonds^18^.

Surface fibrils sticking away from the fiber in dilute suspension entangle during sheet forming and need to be overcome when separating the fibers. Mechanical Interlocking refers to an increase in bond strength by mechanical entanglement between the surfaces; it requires no adhesion force. Another example for mechanical interlocking is bonding under shear load, where the roughness of the interface area contributes to the bond strength. It has been suggested^20^ that mechanical interlocking of the fibrils is the key mechanism in paper strength. Interestingly some interlocking mechanism on the molecular level also seems to take place within the wood fiber cell wall where discrete interlocks are failing sequentially^21^.

**Figure 2:**
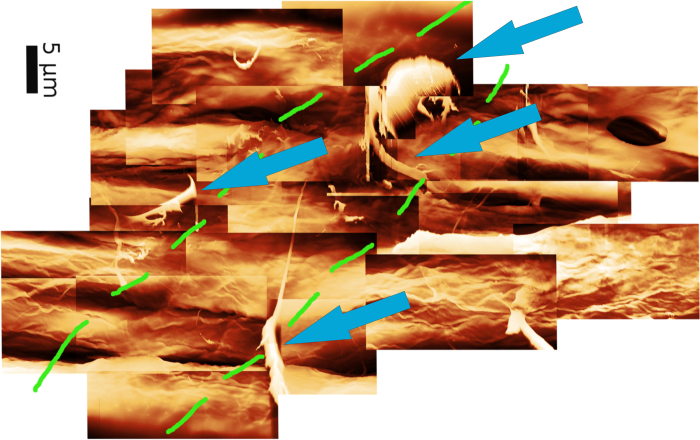
Atomic Force Microscopy image of a formerly bonded area of a fiber-fiber bond reveals the impact of mechanical interlocking. The green dashed lines show the border between the formerly bonded area and the unbonded area. Fibrils extracted out of the fiber (blue arrows) during bond breaking can clearly be seen^22^.

We^22^ and others^23^ observed an effect of external fibrillation on bonding strength. An increase in bonding energy due to mechanical interlocking is likely due to the sequential failure of substructures like fibrils, fibril bundles and fiber wall components, as observed in^22^. Here single fibrils or fibril bundles are torn out of the fibers (Fig. 2), which leads to force discontinuities in the measured force distance curves^22^. This consumes more energy than a more sudden failure of non-fibrillated fiber surfaces.

Experiments measuring force distance curves via AFM and subsequent analysis of the formerly bonded area show that^22^, mechanical interlocking of fibrils in refined pulp attributes a 30–55% increase in bonding energy. Our unrefined pulp has an unfibrillated surface, so we are assuming a 5–20% increase. Including uncertainties from the measurement this leads to 4.9 × 10^−18^ kJ/μm^2^ minimum and 1.7 × 10^−16^ kJ/μm^2^ maximum bond energies. Measurement of bonding energies and bonded area is described in^22^.

**Figure 3:**
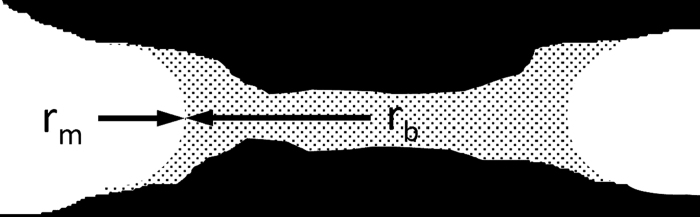
Under ambient conditions, two pulp fiber surfaces are linked by a capillary bridge with the meniscus r_m_ and the radius r_b_. Drying leads to a decrease in the radius r_m_, which will increase the force pressing the two surfaces together leading to a higher bonding energy between the fibers.

Pulp fibers are hygroscopic and contain about 10% water at ambient conditions. Capillary bridges (Fig. 3) are created in the wet state during paper formation^24^. They do not all disappear during drying^10^. Thus, bridges of water between the fiber surfaces create capillary forces in paper.

The meniscus in the capillary bridge (r_m_) is responsible for the Laplace pressure^10^ pulling the surfaces together. The bonding energy per unit area is W/A_0_.
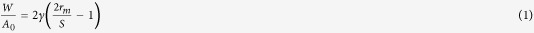


It depends on the water surface tension γ and the initial surface distance 2r_m_. When the surfaces are separated, the capillary bridge collapses at some distance S. For S >>2r_m_, the separation energy according to equation (1) becomes 1.44 × 10^−16^ kJ/μm^2^. This corresponds exactly to the surface energy density necessary to create two new water surfaces making it a plausible value, which is an upper limit for the bonding energy of capillary bridges. For the lower limit a separation distance for collapse of the liquid bridges of 2r_m_/S = 0.1 is assumed, leading to 1.44 × 10^−17^ kJ/μm^2^. In a fiber-fiber bond, the area available for liquid bridges is the region *without* molecular contact. For full molecular contact the contribution of this mechanism drops to zero. For 99.9% molecular contact, the bonding energy constitutes 1.4 × 10^−19^ kJ/μm^2^, while for 30% molecular contact it is 9.8 × 10^−17^ kJ/μm^2^.

Pulp fibers contain carboxyl groups due to the uronic acids in the hemicellulose. These carboxyl groups dissociate in water and are likely to form Coulomb bonds with Na^+^ or Ca^++^ counter ions^25^ during sheet forming. We denote the probability that a carbohydrate monomer on a fiber surface carries a carboxylic group with p(COOH). A first order approximation of the probability that both opposing surfaces have a carboxylic group at the same position is p^2^(COOH). Thus the density of Coulomb interactions between fiber surfaces equals p^2^(COOH) multiplied by the amount of carbohydrate monomers per nm^2^ of fiber surfaces.

**Figure 4:**
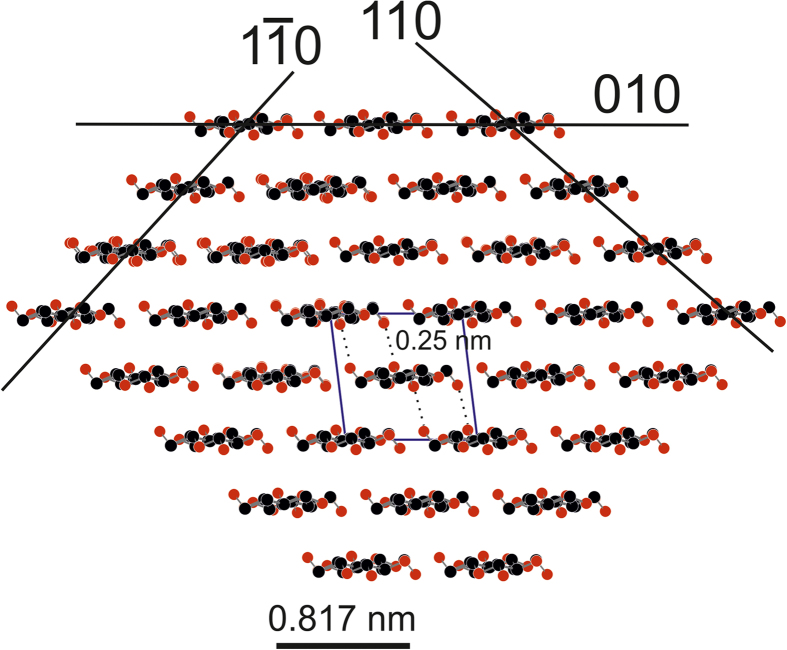
The crystal structure of cellulose I was used to calculate the number of possible Coulomb bonds, hydrogen bonds, and Van der Waals bonds in order to quantify their impact on the bond energy between paper fibers. The black lines show three low index surfaces. Carbon atoms are black, oxygen atoms are red, and hydrogen atoms are not shown. The unit cell is indicated (blue lines) and the distance between two cellulose chains situated in the corners of the unit cell is 0.817 nm, indicated by the bar below the structure. Dashed lines indicate intra-molecular hydrogen bonds in the unit cell. The number of possible hydrogen bonds was calculated from this structure, assuming cleavage along the (010) surface.

X-ray Photoelectron Spectroscopy analysis of unbleached softwood Kraft pulp^26^ gave 10% surface coverage of extractives and a lignin to carbohydrate ratio of 30/70. This leads to a coverage of 63% carbohydrates on the fiber surface. 10% are xylanes^27^ with a ratio of uronic acids to other carbohydrates of 2/11. The probability of a carbohydrate monomer carrying a carboxyl group on the surface thus corresponds to p(COOH) = 0.01145. The number of carbohydrate monomers per surface area is approximated from a cellulose single crystal as shown in Fig. 4. One unit cell containing four monomers covers an area of 0.817 × 1.043 nm^2^ resulting in 4.7 monomers per nm^2^. The Coulomb interaction energy between COO^−^ and Na^+^ is 7536 kJ/mol^28^, which is 1.25 × 10^−20^ kJ per carboxylic group. The bonding energy of Coulomb interactions per μm^2^ of fiber surface is 7.7 × 10^-18^ kJ/μm^2^. This value for the Coulomb related bonding energy varies vastly with changing surface charge due to the quadratic term p^2^(COOH) for bonding probability. We assume that the xylane concentration on the fiber surface is equivalent to the concentration in bulk. Some sources claim that there is a moderate^29^ or massive^30^ depletion of anionic groups on the surface, while others report a moderate increase^31^. Surface depletion of carboxylic groups^30^ leads to a drop to 1.9 × 10^−20^ kJ/μm^2^, which is the lower limit of our estimation. One might also consider a situation where all carboxylic groups on the surface form a Coulomb bond, e.g. by bridging the gap between the oppositely charged surfaces using cationic polymers, which are the most potent strength enhancers in the production of paper^2^. In this case the bonding probability becomes p(COOH) and the bonding energy increases 87-fold (6.6 × 10^−16^ kJ/μm^2^). This value is the upper limit of our estimation.

The crystal structure of cellulose I has an interlayer distance of about 0.207 nm (Fig. 4). The bond length of hydrogen bonds in cellulose I is 0.275 nm^32^. Figure 4 shows that two O atom centers from OH groups point downward in the upper layer and two are pointing upward in the lower layer. The distance is about 0.25 nm. That means that 4 hydrogen bonds between layers can be formed per unit cell (dashed lines). This approximate disctance is within the range of a hydrogen bond length considering that the corresponding hydrogen atoms are not shown in Fig. 4. This leads to approximately 4.7 hydrogen bonds per nm^2^.

An average hydrogen bond energy of 11.63 kJ/mol^33^ results in a bonding energy of 1.9 × 10^−23^ kJ per particle. This provides a hydrogen bonding energy of 8.9 × 10^−17^ kJ/μm^2^. One has to keep in mind that this is an upper limit, as it is derived for a single crystal surface. Haddad^34^ has published a more detailed approach on hydrogen bonding in cellulose. He calculated a maximum number of hydrogen bonds of 6, stating that not all of them are free to form bonds.

Van der Waals interactions are estimated using the crystal structure of cellulose I. The surfaces exposed on the fiber correspond to the (110), (010) and (1–10) surfaces of crystalline cellulose I^35^. Firstly, the amount of atoms on the surface per nm^2^ is calculated. One finds 41 atoms per unit cell on the (010) plane of cellulose I. This number is the sum of carbon, oxygen and hydrogen atoms. With the area of the unit cell of 0.85 nm^2^ one gets approximately 48 atoms per nm^2^.

Secondly, Van der Waals calculations were done using the functional of Perdew Burke and Ernzerhof (PBE)^36^ augmented by the Tkatchenko-Scheffler scheme^37^ to account for the missing long-range Van der Waals forces. For two cellulose molecules, we get a value of 6.8 × 10^−24^ kJ per atom. This compares quite well with the Hamaker constant given in^38^,^39^. With 48 atoms per nm^2^ this bond energy adds up to 3.3 × 10^−16^ kJ/μm^2^. This is significantly larger than the value for hydrogen bonds. Again this is an upper limit, because the bond between two single crystal surfaces has been used for our estimate. The Van der Waals forces within a cellulose microfibril reported in other studies^40^,^41^ are approximately 624 kJ/mol chain depending on the position within the microfibril. With the chain lengths given in^41^,^42^, one achieves the same energy range as estimated here.

The total energy dissipated in breaking fiber-fiber bonds can be evaluated experimentally by cyclic loading and unloading individual fiber-fiber bonds and recording the dissipation energy in each cycle, i.e. the difference between the mechanical energy consumed during loading and the elastic energy released during unloading of the bond. Previously, we performed such experiments for fiber bonds under shear load^42^ and under normal load^22^,^43^. The latter work uses atomic force microscopy (AFM) for cyclic loading and the measurement of the dissipation energy, the experiments were conducted under ambient conditions (20 °C, 40 + −10% RH). This setup is considerably faster, and it involves considerably less deformation of the fibers than shear testing, which reduces the amount of energy consumed by plastic deformation of the fibers. Therefore, the normal load AFM measurements provide dissipation energy values closest to the actual bonding energy. Measurements of dissipated energy for 10 individual fiber-fiber bonds gave a value of 1 × 10^−13^ to 1 × 10^−12^ kJ/bond^22^. Measurement of the optically bonded area for the sample pulp on 89 specimens gave an average bonding area of 1130 μm^2^^44^,^45^ per fiber-fiber bond. Taking our result for the area in molecular contact as 90% of the optically bonded area^6^,^10^ this leads to 1017 μm^2^ that are actually bonded on a molecular level. This gives a measured dissipated energy of 8.9 × 10^−17^ kJ/μm^2^ to 8.9 × 10^−16^ kJ/μm^2^. The dissipation energy contains plastic energy consumed by deformation of the fibers, thus it is an upper bound for bonding energy of the fiber-fiber bond.

## Discussion and Conclusions

**Figure 5:**
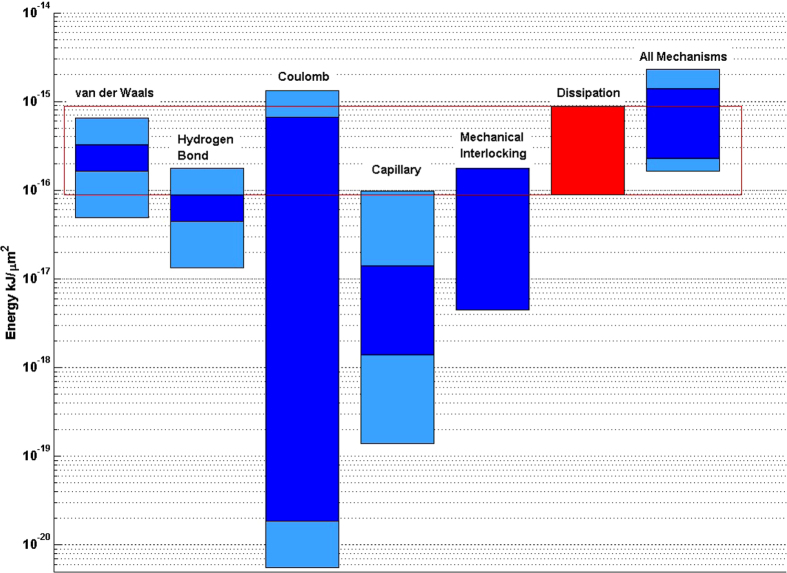
Coulomb and Van der Waals bonds show the highest possible binding energy from all five bonding mechanisms with a large error for Coulomb. The red bar shows the dissipated energy measured for fiber-fiber bonds. Dark Blue are the estimated contributions of different bonding mechanisms without interdiffusion and a fixed area in molecular contact. The light blue parts show the increase in uncertainty from the upper- and lower-limits from interdiffusion and the area in molecular contact.

This work demonstrated experimentally that a large fraction of the fiber-fiber bond in paper is mediated through molecular contacts. This is the starting point for the mechanistic model of fiber bonding presented in this study. It was interesting to find (Fig. 5) that the estimates for the individual bonding mechanisms are fairly close to each other.

One of the main results in this study reveals that the bonding energy estimate for hydrogen bonding, which is the most often cited fiber-fiber bonding mechanism, turns out to be actually one of the less important ones. According to our calculations, Van der Waals interactions are the most prominent, considering the large error bar for Coulomb. This changes the traditional understanding of how fiber-fiber bonding in paper takes place. Due to the high bonding energy of individual Coulomb bonds, this mechanism has the potential to become the dominating contributor for fiber-fiber bonding, as it is relatively facile to add charges to the fiber surface, e.g. by carboxylation.

In a previous study^40^, the authors report the following contributions to the energy gained by forming a microfibril: hydrogen bonding (9.1%), Van der Waals interactions (3.2%) and Coulomb interactions (87.7%). This is comparable to the bond energies estimated here, as^40^ stated that their model underestimates Van der Waals bonds.

Future studies in our laboratory intend to clarify the assumptions that were made here to estimate Coulomb bonding energy. The contribution of capillary bridges is lower than most of the other bonding mechanisms, since capillary forces can only occur in the rather small surface fraction *without* molecular contact. The actual contribution of mechanical interlocking is also rather uncertain. It is likely to be under the maximum value given in Fig. 5. The literature regarding this mechanism is contradictive^22^,^46^. It will be necessary to clarify the contribution of mechanical interlocking to fiber bonding. In addition, the bonding potential for interdiffusion is large^2^,^14^,^16^, but little research dealing with this bonding mechanism has been published. Increased research in this field will bring major insights in the fiber-fiber bonding process.

Importantly, bonding energy does not directly relate to bonding strength. Coulomb bonding has a very high bonding energy, considering that the force-distance relationship is proportional to 1/r^2^ the high bonding energy does not necessarily relate to a particularly high bonding force. Van der Waals interactions on the other hand have lower bonding energies, but considering a force-distance relationship proportional to 1/r^6^ this leads to high bonding forces when the surfaces are proximal. The relationship between dissipation energies and bonding forces are complex for both mechanical interlocking and capillary bridges, as both depend on the topography of the bonded surface. The calculated bonding energy values fit surprisingly well with the dissipated energy measurements on fiber-fiber bonds. The measured dissipated energy is higher than the energy actually consumed to separate the bonded fiber surfaces because of viscoelastic deformation of the pulp fibers. Therefore, our results for the bonding mechanisms are somewhat high. This is due to the assumption that the contact between the surfaces is parallel between monocrystalline cellulose surfaces. In reality, the amorphous structure of hemicellulose and cellulose on the fiber surface will partially inhibit molecular contact, which leads to lower bonding energies.

It is well known that increasing relative humidity reduces paper strength. It is most important to understand that a humidity related drop in paper strength is not necessarily related to a weakening of the fiber-fiber bonding. Network mechanics are playing a pivotal role in paper failure. A key mechanism providing paper strength is the ability of the network to transfer local stress concentrations (e.g. due to faults in the network) to neighboring fibers. This ability to transfer local stress concentrations is, among other mechanisms like straining during drying^47^, heavily influenced by softening of the fibers. As an example tensile strength of bone dry paper drops by factor 3 to 6 when it is tested at different temperatures between −25° to 250 °C^48^. This behavior is caused by thermal softening of the fibers, which are viscoelastic due to their partly amorphous structure. So in conclusion the decrease in paper strength upon increased relative humidity is not necessarily caused by a decrease of fiber-fiber bond strength. It is definitely related to the softening of the fibers upon water uptake and a resulting deterioration in load distribution within the network. If water enters the bonding zone between two fibers it will definitely impair all fiber-fiber bonding mechanisms except capillary bridges which might improve and hydrogen bonds which may bridge over a thin layer of water . But we can only speculate about the critical relative humidity when water is actually entering the bonding zone. Thus, at this point, we can not answer the question if and how changes in relative humidity affects fiber-fiber bonding.

The present work is an initial step into quantitative analysis of fiber-fiber bonding mechanisms. Our analysis revised the prevalent notion of hydrogen bonding as the most relevant bonding mechanism. Instead, our results identified Van der Waals forces, Coulomb bonding, and the extent of molecular contact as the key factors for fiber-fiber bonding. This study provides a step towards answering the old question of what is actually holding paper fibers together. In the interest of reducing the large amounts of wood consumption and energy required in papermaking, the comprehensive quantitative understanding of the fundamental interactions in paper making could lead to advances in both research and industrial applications, while reducing both financial cost and environmental impact.

## Additional Information

**How to cite this article**: Hirn, U. & Schennach, R. Comprehensive analysis of individual pulp fiber bonds quantifies the mechanisms of fiber bonding in paper. *Sci. Rep.* **5**, 10503; doi: 10.1038/srep10503 (2015).

## References

[b1] Rainey T. J. Doherty W. O. S., Martinez D. M., Brown R. & Kelson N. A. Pressure Filtration of Australian Bagasse Pulp, Transport in Porous Media , 86, 737–751 (2010).

[b2] Hubbe M. Bonding between cellulosic fibers in the absence and presence of dry-strength agents-A review. BioResources 1, 281–318 (2006).

[b3] Lindström T., Wågberg L. & Larsson T. *On the Nature of Joint Strength in Paper – A Review of Dry and Wet Strength Resins used in Paper Manufacutring*, in Advances in Paper Science and Technology, Trans. 13^th^ Fund. Res. Symp. Cambridge, 457–562, Cambridge, FRC (2005).

[b4] Page D. A theory for the tensile strength of paper, Tappi J. 52, 674–681 (1969).

[b5] Rennel J. Opacity in Relation to Strength Properties of Pulp; Part 4–The Effect of Beating and Wet Pressing, Pulp and Paper Canada 70, T151–T158 (1969).

[b6] Hirn U., Schennach R., Ganser C., Magnusson M., Teichert C. & Östlund S. The area in molecular contact in fiber-fiber bonds , in Advances in Paper Research, Trans. 15^th^ Fund. Res. Symp. Cambridge, 201–226, Cambridge, FRC (2013).

[b7] Wågberg L. & Annergren G. Physico-chemical characterization of papermaking fibers , in The Fundamentals of Papermaking, Trans. 11^th^ Fund. Res. Symp. Cambridge, 1–82, Cambridge, FRC (1997).

[b8] Campbell W. B. The Mechanism of Bonding, Tappi J. 42, 999–1001 (1959)

[b9] Persson B. N. J. Capillary adhesion between elastic solids with randomly rough surfaces, J. Phys.: Condens. Matter 20, 315007(11p) (2008).

[b10] Persson B. N. J., Ganser C., Schmied F., Teichert C., Schennach R., Gilli E. & Hirn U. Adhesion of cellulose fibers in paper, J. Phys.: Condens. Matter 25, 045002(11p) (2013). 23220767 10.1088/0953-8984/25/4/045002

[b11] Yang C. & Persson B. N. J. Contact mechanics: contact area and interfacial separation from small contact to full contact, J. Phys.: Condens. Matter 20, 215214(13pp) (2008). 10.1103/PhysRevLett.100.024303 18232875

[b12] Ganser C., Hirn U., Rohm R., Schennach R. & Teichert C. AFM nanoindentation of pulp fibers and thin cellulose films at varying relative humidity, Holzforschung , 68, 53–60 (2014).

[b13] Chhabra N., Spelt J. K., Yip C. M. & Kortschot M. T. An investigation of pulp fibre surfaces by atomic force microscopy, J. Pulp Paper Sci. 31, 52–56 (2005).

[b14] Pelton R. A model of the external surface of wood pulp fibers, Nord. Pulp Pap. Res. J. 8, 113–119 (1993).

[b15] Krause T. & Schrempp W. Sheet Forming - Theoretical Aspects and Practical Experiences, Wochenblatt der Papierfabrikation 106, 21–27 (1978).

[b16] McKenzie A. The structure and properties of paper. Part XXl: The diffusion theory of adhesion applied to interfibre bonding, Appita J. 37, 580–583 (1984).

[b17] Thomson C., Lowe R. & Ragauskas A. Imaging celulose fibre interfaces with fluorescence microscopy and resonance energy transfer, Carbohydrate Polym. 69, 799–804 (2007).

[b18] Thomson C., Lowe R. & Ragauskas A. First characterization of the development of bleached kratf softwood pulp fiber interfaces during drying and rewetting using FRET technology, Holzforschung 62, 383–388 (2008).

[b19] Thomson C., Lowe R., Page D. & Ragauskas A. Exploring fibre-fibre interfaces via FRET and fluorescence microscopy, J. Pulp and Paper Sc . 34, 113–119 (2008).

[b20] Linhart F. Some thoughts on the mode of action of paper strength agents, Wochenblatt der Papierfabrikation 133, 662–672 (2005).

[b21] Kretschmann D., Velcro mechanics in wood, Nature Materials 2, 775–776 (2003). 14647489 10.1038/nmat1025

[b22] Schmied F. J., Teichert C., Kappel L., Hirn U., Bauer W. & Schennach R. What holds paper together: Nanometre scale exploration of bonding between paper fibres, Scientific Reports 3, 2432(10p) (2013). 23969946 10.1038/srep02432 PMC3749796

[b23] Kang T. & Paulapuro H. Effect of External Fibrillation on Paper Strength, Pulp and Paper Canada 107, 51–54 (2006).

[b24] Van de Ven T. G. M. Capillary forces in wet paper, Ind. Eng. Chem. Res. 47, 7250–7256 (2008).

[b25] Rohm S., Hirn U., Ganser C., Teichert C. & Schennach R. Thin cellulose films as a model system for paper fibre bonds, Cellulose 21, 237–249 (2014).

[b26] Wågberg L. in Paper Chemistry and Technology Vol. 3, Ch. 4, 65–92, (de Gruyter, 2009).

[b27] Aln R. in Book 3-Forest Products Chemistry, Ch. 1, 1–57, (Fapet Oy, 2000).

[b28] Bendiksen B., Friberg S. & Plummer P. M., CNDO Calculations on the Structure of a Liquid-Sodium Carboxylate-Carboxylic Acid Compound J. Coll. Interf. Sci. 72, 495–504 (1979).

[b29] Barzyk D., Page D. & Ragauskas A. Acidic Group Topochemistry and Fiber-to-Fiber Specific Bond Strength, J. Pulp Paper Sc . 23, J59–J61 (1997).

[b30] Fardim B. H. P. & Holmbom B. Origin and Surface Distribution of Anionic Groups in Different Papermaking Fibers, Coll. Surf. A 252, 237–242 (2005).

[b31] Sjöberg J., Kleen M., Dahlman O., Agnemo R. & Sundvall H. Analyses of carbohydrates and lignin in the surface and inner layers of sotfwood pulp fibers obtained employing various alkaline cooking processes Nordic Pulp & Paper Research Journal 17, 295–301 (2002).

[b32] O’Sullivan A. Cellulose: the structure slowly unravels, Cellulose 4, 173–207 (1997).

[b33] Oliveira B. G., Pereira F., de Arajo R. & Ramos M. The hydrogen bond strength: New proposals to evaluate the intermolecular interaction using DFT calculations and the AIM theory, Chem. Phys. Lett. 427, 181–184 (2006).

[b34] Haddad Y. & Theoretical A Approach to Interfiber Bonding of Cellulose, J. Coll. Interf. Sci . 76, 490–501 (1980).

[b35] Ding S. Y. & Himmel M. E. The maize primary cell wall microfibril: a new model derived from direct visualization, J. Agri. and Food Chem. 54, 597–606 (2006). 10.1021/jf051851z 16448156

[b36] Perdew J. P., Burke K. & Ernzerhof. M. Generalized Gradient Approximation Made Simple, Phys. Rev. Lett . 77, 3865–3868 (1996). 10062328 10.1103/PhysRevLett.77.3865

[b37] Tkatchenko A. & Scheffler M. Accurate Molecular Van Der Waals Interactions from Ground-State Electron Density and Free-Atom Reference Data, Phys. Rev. Lett. 102, 073005(4p) (2009). 19257665 10.1103/PhysRevLett.102.073005

[b38] Notley S. & Wågberg L. *Direct measurement of attractive Van der Waals Forces and repulsive electrostatic forces between regenerated Cellulose surfaces in an aqueous environment*, in Advances in Paper Science and Technology, Trans. 13^th^ Fund. Res. Symp. Cambridge, 1337–1350, Cambridge, FRC (2005).

[b39] Notley S., Petersson B. & Wågberg L. Direct Measurement of Attractive van der Waals’ Forces between Regenerated Cellulose Surfaces in an Aqueous Environment J. Am. Chem. Soc. 126, 13930–13931 (2004). 15506747 10.1021/ja045992d

[b40] Baird M., O’Sullivan A. & Banks W. A native cellulose microfibril model, Cellulose 5, 89–111 (1998).

[b41] Cousins S. & Brown R., Cellulose I microfibril assembly: computational molecular mechanics energy analysis favours bonding by van der Waals forces as the initial step in crystallization, Polymer 36, 3885–3888 (1995).

[b42] Fischer W. J., Hirn U., Schennach R. & Bauer W. Testing of individual fiber-fiber joints under biaxial load and simultaneous analysis of deformation, Nordic Pulp & Paper Research Journal 27, 237–244 (2012).

[b43] Schmied F., Teichert C., Kappel L., Schennach R. & Hirn U. Joint strength measurements of individual fiber-fiber bonds: An atomic force microscopy based method, Rev. Sci. Inst. 83, 073902(8p) (2012). 10.1063/1.4731010 22852699

[b44] Kappel L., Hirn U., Bauer W. & Schenach R. A novel method for the determination of bonded area of individual fiber-fiber bonds, Nordic Pulp & Paper Research Journal 24, 199–205 (2009).

[b45] Kappel L. Development and application of a method for fiber- fiber bonded area measurement, Ph.D. thesis, Graz University of Technology (2010).

[b46] Mayhood C., Kallmes O. & Cauley M. The mechanical properties of paper – part II: Measured shear strength of individual fiber to fiber contacts, Tappi 45, 69–73 (1962).

[b47] Mäkelä P. *Effect of drying conditions on the tensile properties of paper*, in Advances in Pulp and Paper Science Research, Trans. 14^th^ Fund. Res. Symp. Oxford, 1079-1094, Manchester, FRC (2009).

[b48] Salmen L. & Back E. Effect of temperature on stress strain properties of fibers, Svensk Papperstidning 81, 341–346, (1978).

